# Randomised, double-blind, placebo-controlled clinical trial for evaluating the efficacy of intracoronary injection of autologous bone marrow mononuclear cells in the improvement of the ventricular function in patients with idiopathic dilated myocardiopathy: a study protocol

**DOI:** 10.1186/s12872-019-1182-4

**Published:** 2019-08-22

**Authors:** Miguel Romero, José Suárez-de-Lezo, Concha Herrera, Manuel Pan, José López-Aguilera, José Suárez-de-Lezo, Flor Baeza-Garzón, Francisco Javier Hidalgo-Lesmes, Olga Fernández-López, Juliana Martínez-Atienza, Eva Cebrián, Vanesa Martín-Palanco, Rosario Jiménez-Moreno, Rosario Gutiérrez-Fernández, Sonia Nogueras, Maria Dolores Carmona, Soledad Ojeda, Natividad Cuende, Rosario Mata

**Affiliations:** 1 Cardiology Unit, Reina Sofía University Hospital, Córdoba, Spain; 2 Cell Production Unit, Reina Sofía University Hospital, Córdoba, Spain; 3 Andalusian Initiative for Advanced Therapies, Andalusian Public Foundation Progress and Health - Junta de Andalucía, 41006, Esquina Avda. Hytasa, Seville, Spain

**Keywords:** Dilated myocardiopathy, Randomized controlled trial, Bone marrow mononuclear cells, Cell therapy

## Abstract

**Background:**

Cellular therapies have been increasingly applied to diverse human diseases. Intracoronary infusion of bone marrow-derived mononuclear cells (BMMNC) has demonstrated to improve ventricular function after acute myocardial infarction. However, less information is available about the role of BMMNC therapy for the treatment of dilated myocardiopathies (DCs) of non-ischemic origin. This article presents the methodological description of a study aimed at investigating the efficacy of intracoronary injection of autologous BMMNCs in the improvement of the ventricular function of patients with DC.

**Methods:**

This randomised, placebo-controlled, double-blinded phase IIb clinical trial compares the improvement on ventricular function (measured by the changes on the ejection fraction) of patients receiving the conventional treatment for DC in combination with a single dose of an intracoronary infusion of BMMNCs, with the functional recovery of patients receiving placebo plus conventional treatment. Patients assigned to both treatment groups are monitored for 24 months. This clinical trial is powered enough to detect a change in Left Ventricular Ejection Fraction (LVEF) equal to or greater than 9%, although an interim analysis is planned to re-calculate sample size.

**Discussion:**

The study protocol was approved by the Andalusian Coordinating Ethics Committee for Biomedical Research (*Comité Coordinador de Ética en Investigación Biomédica de Andalucia*), the Spanish Medicines and Medical Devices Agency (*Agencia Española de Medicamentos y Productos Sanitarios*), and is registered at the EU Clinical Trials Register (EudraCT: 2013–002015-98). The publication of the trial results in scientific journals will be performed in accordance with the applicable regulations and guidelines to clinical trials.

**Trial registration:**

ClinicalTrials.gov Identifier NCT02033278 (First Posted January 10, 2014): https://clinicaltrials.gov/ct2/show/NCT02033278; EudraCT number: 2013–002015-98, EU CT Register: https://www.clinicaltrialsregister.eu/ctr-search/search?query=2013-002015-98. Trial results will also be published according to the CONSORT statement at conferences and reported peer-reviewed journals.

## Background

### Background and rationale

In the last few years we have assisted to a large increase on the use of cellular therapies, propelled by the identification of stem cells with regenerative properties in virtually all adult tissues. This has been particularly notorious in the field of cardiology, probably due to the high prevalence of cardiac diseases and the easy monitorization of cardiac function.

Recent research in the field has changed the concept of the heart as an organ exclusively constituted by postmitotic cells and, therefore, unable to regenerate. According to this dogma maintained for decades, the number of cardiac myocytes each individual is born with, is maintained throughout their life and if some myocytes dies (for example, as a result of a heart attack) they cannot be replaced in any way. Initial observations by Anversa et al. [1] seriously questioned this concept by demonstrating the capacity for myocardial regeneration from resident cells after acute myocardial infarction (AMI). Nowadays, several randomized studies and meta-analyses demonstrate a significant improvement in post-AMI ventricular function following intracoronary infusion of autologous bone marrow-derived mononuclear cells (BMMNC) [2–10]. Even though the role of cellular therapy in the treatment of ischemic heart disease has been extensively studied [11], its potential application to other causes of congestive heart failure that still constitute an important cause of cardiovascular mortality has not been explored in depth. Such is the case of dilated myocardiopathies (DCs) of non-ischemic origin, which constitute a major cause of heart failure. This group of primary myocardial diseases is characterized by a loss of cardiomyocytes along with an increase of fibroblasts in cardiac tissue. Therefore, restoring cardiomyocytes using autologous bone marrow (BM) cells could constitute an effective therapy for DC, improving cardiac function.

Different experimental studies in animal models of DC have shown improvements in ejection fraction and decreases in the ventricular volumes after cell therapy [12–21]. These benefits have also been established in clinical studies aimed at analysing the safety and efficacy of bone marrow-derived progenitor cells in patients with DC [22–31]. In 2006, the ABCD trial [26] described for the first time in humans improvements in the NYHA functional class, a decrease in end-diastolic volume and an average improvement of 5.4% in the ejection fraction in 24 patients with DC treated with intracoronary BM-derived cells. A long term follow-up of this trial showed that the functional benefits achieved were maintained without late adverse effects [25]. Similarly, Fischer-Rasokat et al. [23] observed in 33 patients with DC that intracoronary administration of BM-derived cells appears to be associated with improvements in cardiac contractile and microvascular function in patients with DC (ref). More recently, these results have been confirmed by the REGENERATE-DCM clinical trial [30], in which a 5.37% increase in left ventricular ejection fraction (LVEF) was observed at 3 months after treatment with autologous BM-derived cells combined with peripheral granulocyte colony-stimulating factor (G-CSF). Next to these previous experiences, our group has completed a one-year Phase II Clinical Trial (NCT00629096, EudraCT 2007–003088-36) to evaluate the safety and efficacy of intracoronary infusion of autologous BMMNCs in 27 patients with DC [32]. In this study, an improvement in the New York Heart Association (NYHA) functional class and a reduction of brain natriuretic peptide (BNP) plasma levels were observed without any adverse effect. In addition, a significant decrease in ventricular volumes along with a mean increase in the ejection fraction of 9% in 20 out of the 27 patients was observed at month 6. These changes suggest a favourable effect of cell therapy on ventricular remodelling, which may explain the decrease (or even disappearance) of the degree of mitral regurgitation observed in some patients. However, the gain in ejection fraction after cell therapy varied greatly between patients, ranging from − 9% to + 34%, being this gain greater in patients with a baseline ejection fraction < 25%. The clinical benefit of cell therapy was stable 5 years after infusion, with 69% survival rate and 43% of patients free of major events, staying within a NYHA functional class of I-II [33]. Interestingly, younger patients seemed to show the highest long term response to cell-based therapy in terms of LVEF improvement. However, contradictory reports have been published which show no improvements in ejection fraction, NYHA functional class, or exercise test outcomes after intracoronary infusion of autologous BMMNCs in patients with DC [29]. However these findings should be interpreted with caution as, despite apparent similarities, important methodological differences exist among published reports: BMMNCs dose, LVEF measuring technique, administration procedure, clinical description of eligible patients, subject follow-up length, use of G-CSF, technique of bone marrow cell harvesting etc. Ultimately, clinical studies using BM-derived cells in DC patients have shown only modest benefits [34–36]. We believe that the proposed clinical study may contribute with a new methodological perspective on search for cell therapy-based treatment alternatives in patients with DC. In summary, there is sufficient preliminary evidence to consider intracoronary injection of BMMNCs as a viable, safe and beneficial treatment in patients with DC. However, there is a need for larger, randomized, controlled studies to understand the biological mechanism of action of BMMNCs in the myocardium and the factors affecting clinical response to regenerative therapy with autologous BMMNCs in patients with DC.

### Objectives

This article presents and discusses the methodological description of a randomized, placebo-controlled, double-blinded phase IIb clinical trial aimed at evaluating the efficacy of intracoronary injection of autologous BMMNCs in improving ventricular function among patients with idiopathic DC, for which there is currently no effective therapeutic alternative. The secondary objectives of the study are: to analyse the clinical, functional and biological factors that predict a good response to the BMMNC therapy; as well as to confirm the safety of the product and the route of administration.

## Methods and design

### Trial design

**Fig. 1:**
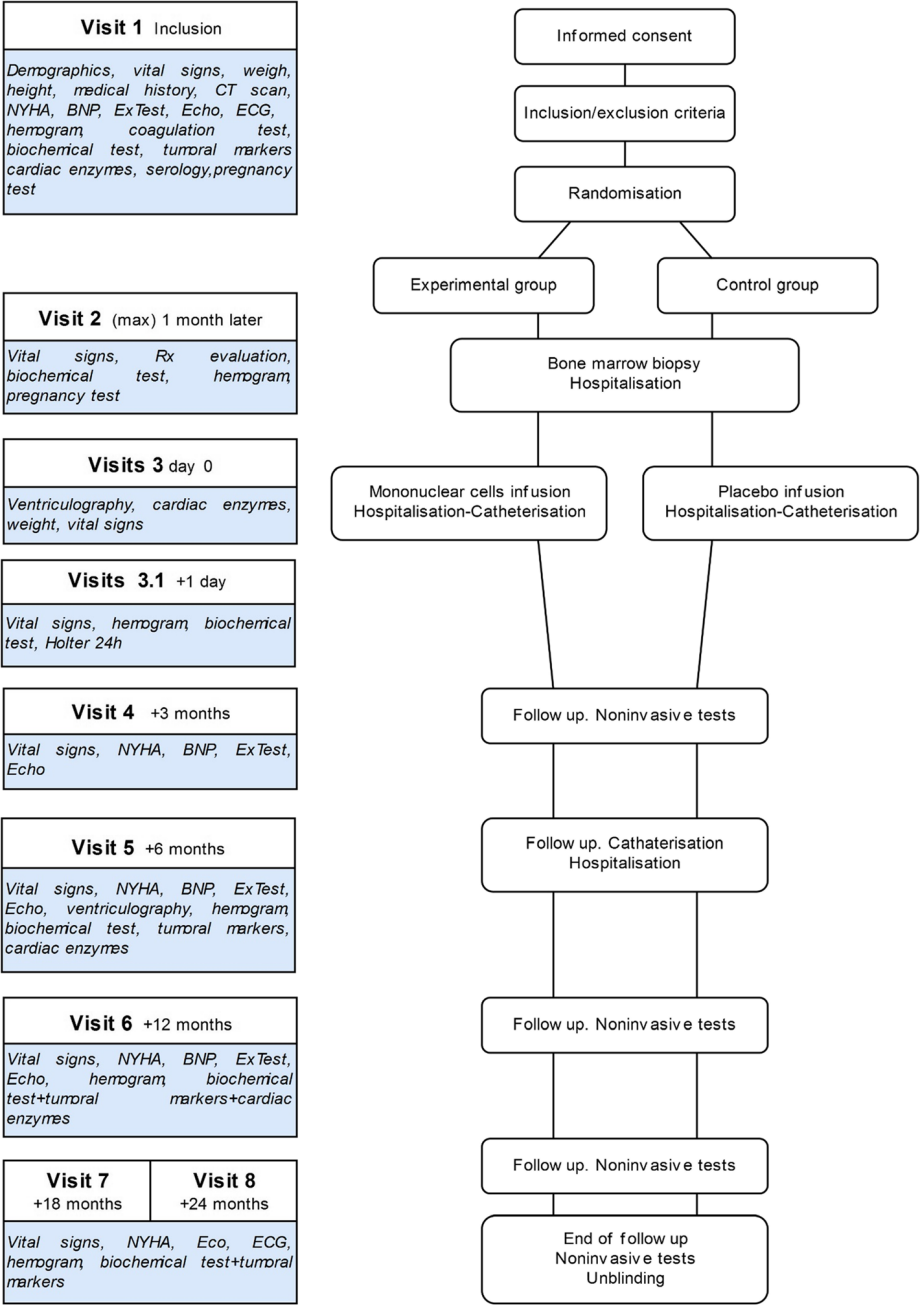
Study design and assessment timeline. Clinical trial visits are structured in 9 time-points, that include two pre-infusion visits (screening and randomization), BMMNC/placebo infusion day, and 6 post-infusion follow-up evaluation time-points according to a decreasing frequency: 24 h, 3 months, 6 months, 12 months, 18 months and 24 months. The procedures and evaluations performed are detailed for each visit. Biochemical determinations: glucose, urea, creatinine, sodium, potassium, C reactive protein (CRP), aspartate aminotransferase (AST), alanine aminotransferase (ALT), gamma-glutamyl transferase (GGT), alkaline phosphatase (ALP), bilirubin, total cholesterol, HDL-cholesterol, LDL-cholesterol, triglycerides, lactate dehydrogenase (LDH). Cardiac enzymes: Creatine kinase (CK), Troponin I or US and brain natriuretic peptide (BNP) or N-terminal fragment of pro-brain natriuretic peptide (NT-proBNP). Demographic data: date of born, sex. Echocardiogram (Echo): Left ventricular (LV) ejection fraction (LVEF, %), LV end-diastolic volume (LVEDV, ml), LV end-systolic volume (LVESV, ml), LV end-diastolic diameter (LVED, mm), LV end-systolic diameter (LVSD, mm), left atrial (LA) area (cm2), right ventricular (RV) end-diastolic volume (RVEDV, ml), Tricuspid annular plane systolic excursion (TAPSE, mm). Electrocardiogram (ECG): synusal rhythm, conduction disturbances, atrial fibrillation (AF), right bundle branch block (RBBB), left bundle branch block (LBBB), left anterior hemiblock (LAHB), QRS duration (sec), PR duration (sec), heart rate (bpm). Exercise testing (ExTest) (according to Naughton protocol): Metabolic equivalents (METS), exercise time (min). Hemogram: red blood cell (RBC) count, hemoglobin, hematocrit, white blood cell (WBC) count (neutrophils, lymphocytes, monocytes, eosinophils, and basophils), erythrocyte sedimentation rate (ESR), platelets. Medical history: personal antecedents, cardiovascular risk factors (hypertension, dyslipidemia, tobacco and alcohol consumption, history of ischemic cardiomyopathy). Clinical evaluation: adverse events (AEs), concomitant drugs and procedures evaluation. Serology: human immunodeficiency virus (HIV), Hepatitis B virus (HBV), Hepatitis C virus (HCV). Tumoral markers: CA125, CA19.9, carcinoembryonic antigen (CEA), and alpha-fetoprotein (AFP), Beta-hCG (BHCG), prostate-specific antigen (PSA). Ventriculography: LVEF in sinus rhythm (%), LVEF in sinus rhythm post-PVC (premature ventricular contraction) (%), Sinus end-diastolic volume (mL/m2), Sinus end-systolic volume (mL/m2), Post-PVC end-diastolic volume (mL/m2), Post-PVC end-systolic volume (mL/m2), hypokinesia in sinus rhythm, hypokinesia post-PVC, acute coronary syndrome (ACS) in sinus rhythm, ACS post-PVC, left ventricular end-diastolic pressure and heart rate (bpm). Vital signs: blood pressure (mmHg), heart rate (bpm)

The study is designed as randomized, placebo-controlled, double-blind phase IIb clinical trial to compare the functional recovery of patients receiving the conventional treatment for DC in combination with an intracoronary infusion of BMMNCs, using placebo as a control. A 2:1 BMMNC/placebo allocation ratio is used for the randomization of a total of 51 eligible patients. Therefore, a total of 34 subjects will receive BMMNCs infusion, whereas 17 subjects will be treated with a placebo infusion. In order to analyse the predictors of clinical response as well as safety variables, all trial subjects (*n* = 51) are followed for a period of 24 months, completing a total of nine study visits: screening (visit 1), randomization (visit 2), infusion (visit 3), 24 post-infusion (visit 3.1), and months 3 (visit 4), 6 (visit 5), 12 (visit 6), 18 (visit 7) and 24 (visit 8) after the BMMNC/placebo administration (Fig. 1). To allow for sample size re-calculation an interim analysis will be performed with the data from the 6 months post-infusion period of the first 20 patients included. A second interim analysis is planned when the last patient included in the trial has completed study visit 5 (6 months). This early analysis of the data may provide conclusive efficacy (and safety) evidence in order to allow for the authorization of compassionate treatment of finalized patients allocated to placebo. On-completion of protocol standardized 24 months’ follow-up, all patients receiving the experimental treatment will be followed on an observational scheme for an additional period of up to 5 years. This extended follow-up period will provide long term efficacy and safety data of BMMNCs infusion.

This is an investigator-driven clinical trial lead by an team of cardiologist and hematologist, and sponsored by the Andalusian Initiative for Advanced Therapies (AIAT), through the Andalusian Public Foundation Progress and Health, acting as a coordinating and advisory entity to help with the complex regulatory requirements needed for clinical implementation of basic research findings [37]. AIAT is also responsible for handling clinical trial administrative authorizations and regulatory affairs, providing study drug and supplies, independent data and safety monitoring, centralised manufacturing supervision, as well as general coordination and daily operational management of the trial for all of the participating sites. Patients or public where not involved in the design, conduct or recruitment of the trial, yet trial results will be disseminated to the public through the EU Database, as well as patient’s congresses wherever possible.

### Study outcomes

The main study variable is the improvement on the ventricular function measured angiographicaly as described below. This efficacy outcome is measured as the changes on the LVEF evaluated by a cardiologist blinded to the patients’ clinical follow-up. With this objective, patients undergo cardiac catheterization in two study timepoints: on the date of the intracoronary infusion of either experimental treatment or placebo (visit 3) and at month 6 (visit 5) after infusion.

Secondary efficacy outcomes include clinical evaluation and NYHA functional grade, BNP or N-terminal fragment of pro-brain natriuretic peptide (NT-proBNP) blood levels, echocardiogram, exercise testing according to Naughton protocol, without oxygen consumption as well as biological parameters of the BMMNC product that may predict a good response (cellularity, chemotaxis and stem cell markers: CD133+, CD34+, CD34+/CD177+,CD34+/CD38-, CD34+/CXCR4+, CD34+/DR-, VEGFR2+, CD90+, CD105+, CD73+, CD14-, CD45-).

Study variables regarding safety are measured as appropriate during follow-up visits: adverse events (AEs) and serious adverse events (SAEs), causally related with study interventions or procedures (including marked changes of vital signs, abnormalities physical examinations and out of range laboratory test results). Additionally, the protocol emphasizes the identification of specific adverse events: major cardiac events or MACEs (defined as cardiac death, major arrhythmia, inclusion in heart transplant waiting list, cardiac resynchronization therapy interventions and hospital admission due to clinically relevant adverse cardiac events), symptoms and signs of arrhythmia, evidence of myocardial harm and tumoral biomarkers abnormalities.

The study design, evaluations and assessment timeline for all variables is illustrated in Fig. 1. After product infusion, all patients are followed-up for 24 months according to protocol by interventional or clinical cardiologists at the participating hospitals. Protocol programmed visits are structured in 9 time-points that include two pre-infusion visits (screening and randomization), BMMNC/placebo infusion day, and 6 post-infusion follow-up evaluation time-points according to a decreasing frequency: 24 h, 3 months, 6 months, 12 months, 18 months and 24 months. Trial investigators must ensure that essential clinical, analytical and exploratory data is registered in the study’s Case Report Form (CRF).

### Study population and enrolment

The study population is constituted by adult male and female patients diagnosed with idiopathic DC, meeting all eligibility criteria as described in Table 1. A sample size of 51 subjects who are regularly followed-up in the Cardiology Units of the eight participating University Hospitals from the Andalusian Public Health System (Table 2) are included in the study through a competitive recruitment scheme. Before undergoing any trial-related procedures, all patients willing to participate in the study must sign a written informed consent. Study practitioners provide them with information about the study, the investigational product, the purpose, procedures and potential risks and benefits of the study. They are randomly assigned to the experimental or control group following a 2:1 ratio, and are engaged to complete study follow-up visits through an optimised medical attention. Trial subjects can withdraw consent for trial participation at any time. Once the patient has received treatment, withdrawal of consent is the single exclusion criteria considered by the protocol. In all other cases the patient must complete follow-up visits to meet at least safety objectives. In addition, patients who have not received the investigational product infusion can also leave the study when a SAE occurs, or the clinical situation of the patient changes and the investigator considers that an alternative treatment is needed.

**Table 1:** Eligibility criteria

INCLUSION CRITERIA	
*Participants meeting all the following criteria will be included:*	
1. Male and female patients between 18 and 70 years old.	
2. Patients who sign the informed consent.	
3. Patient diagnosed with idiopathic dilated cardiomyopathy (using echocardiography).	
4. At least 6 months since dilated cardiomyopathy diagnosis.	
5. Lack of coronary injuries on multi-slice computerized axial tomography scan and/or hemodynamic study performed after the inclusion of the patient in the trial, or prior to recruitment if the patient does not present any angina symptomatology.	
6. Patients under optimised pharmacological treatment for at least 6 months prior inclusion in the trial.	
7. Left ventricular ejection fraction (LVEF) ≤ 40% or LVEF 40–50% if left ventricular end diastolic volume (LVEDV) > 110 ml/m^2^ (measured by echocardiogram).	
8. Presence of sinus rhythm.	
9. Normal levels of analytic indicators. Defined by: Leukocytes ≥3000c cel/mm^3^; Neutrophils ≥1500 cel/mm^3^; Platelets ≥100,000 cel/mm^3^; AST/ALT ≤2.5 institution standard range; Creatinine ≤2.5 mg/dl.	
10. Woman of childbearing age with a negative result on a pregnancy test performed at the time of the inclusion and committed to use contraceptive methods during the study.	
EXCLUSION CRITERIA	
*Participants meeting one or more of the following criteria will be excluded.*	
1. Secondary dilated cardiomyopathy	
2. Recent (6 months) myocarditis episodes prior to signing the informed consent.	
3. Patients susceptible to resynchronization therapy (QRS > 150, presenting symptoms and with no response to medication). However non-responders to resynchronization therapy within 6 months may be included.	
4. Patients in the waiting list for cardiac transplantation.	
5. Coexistence of any type of hematologic disorder, malignant or premalignant tumor, or other severe systemic diseases.	
6. Woman of childbearing age not using contraceptives, pregnant or in breastfeeding period.	
7. Patients currently taking part in another clinical trial or those who did so in the last 3 months or those who have participated in a clinical trial with advanced therapy at any time.	
8. Positive serology for hepatitis B, hepatitis C or human immunodeficiency virus.	
9. Patients taking medicines prohibited by protocol at the time of inclusion. A wash-out period of 2 months is established to be eligible for inclusion.

**Table 2:** Study sites and enrolment distribution until December 2018

Clinical Trial Sites^a^	Enrolment
University Hospital Reina Sofía (Córdoba)	24
University Regional Hospital of Málaga	1
University Hospital Virgen del Rocío (Seville)	2
University Hospital Puerta del Mar (Cádiz)	0
University Hospital Virgen de las Nieves (Granada)	1
Hospital Costa del Sol (Marbella)	0
University Hospital Juan Ramón Jimenez (Huelva)	0
University Hospital of Jerez (Jerez de la Frontera)	0
University Hospital Virgen Macarena (Seville)	0
PARTICIPATING HOSPITALS = 9	28 SUBJECTS

^a^Participating hospitals may be subject to eventual changes as restrictive selection criteria and the low prevalence of this disease may demand flexible re-distribution of clinical trial sites in order to to complete the sample size

### Trial status

The first patient was included in February 2014, after the approval of the institutional Ethics Committees and the Spanish Medicines and Medical Devices Agency (*Agencia Española de Medicamentos y Productos Sanitarios, or AEMPS*), and is currently ongoing in nine Spanish hospitals (Table 2). At the time of manuscript submission patient recruitment is active with 27 patients having received the investigational medicinal product so far (53% of trial sample size, *n* = 51). The study will end when the trial’s sample size (*n* = 20) has been completed and the last patient recruited completes a 24 months’ follow-up period.

### Manufacturing of BMMNCs/placebo

Following informed consent all patients undergo a bone marrow extraction through repeated aspirations from the posterior iliac crest to a total volume of 100–180 mL using 5 mL syringes (20–30 aspirations) that will be collected in an sterile plastic bag containing ACD-A (1:5 v/v proportion). The entire extraction procedure lapses 20–30 min. BMMNCs are obtained by density gradient centrifugation over Ficoll-Hypaque in an automated cell processor SEPAX (Biosafe). After two washes with a 4% albumin solution, a 50 ml cell suspension will be filtered (50 μm diameter), and aliquots used for cell count, viability tests, microbiological assays and biological determinations (immune phenotype and chemotactic ability tests). From this point onwards, the procedure proceeds as follows: For the experimental group, the cell suspension undergoes a final centrifugation, is re-suspended in 20 ml of lactate Ringer’s solution (containing 1% human albumin and 2.5% glucose) and filtered by a 50 μm filter. The total dose of BMMNCs is within the range of 5-10 × 10^8^ cells. Cell expansion is not used in this protocol. In our previous pilot experience, the minimum percentage of BMMNC recovery after processing was around 20–30% of the total initial cell count, which is enough to obtain a final cellular number in the order of 5–10 × 10^8^ to be infused. Should the number of cells harvested in the final product be insufficient, patient’s data would be excluded from the efficacy analysis. Cell viability is > 95% of BMMNCs, and this concentration is maintained for 24 h at a temperature between 4 °C and 22 °C degrees centigrade, as it has been validated in our laboratory. For the control group, placebo consists of 20 ml of lactate Ringer’s solution (containing 1% human albumin and 2.5% glucose), plus the required volume of red blood cells from the extracted bone marrow, to adjust the hematocrit to 1% for masking purposes. The pre-processed BMMNCs from patients in the control group is re-suspended in a solution containing 4% albumin and 10% dimethylsulfoxide and cryopreserved for future administration via compassionated use (if efficacy data is conclusive after trial interim/final analysis). The autologous BMMNCs or placebo is placed in 2–3 sterile syringes (depending on coronary artery dominance) and is transported to the cardiac units in the participating sites for the infusion procedure.

### BMMNCs infusion

The administration of placebo or cell product is performed within 6–8 h after product processing. A 10 mL volume is delivered in a single dose through the three main coronary arteries: anterior descending, circumflex and right coronary artery. Fifty percent of the dose is administered through the anterior descending artery and the other 50% through the right or circumflex coronary artery, depending on the dominance. If a clear dominance is absent, 25% is administered through the right artery, and 25% through the circumflex artery. Exceptionally, in case of hypoplasia of the descending artery, the product is administered in the same proportions (33%) though the three coronary arteries.

### Angiographic measurement of ventricular function

Angiographic changes in global LVEF at 6 months after cell infusion is the primary endpoint of the trial. Cardiac catheterization is performed to measure systemic and left ventricular end-diastolic pressure, left ventricular function, and coronary flow reserve for each coronary artery as previously published [32]. The left ventricular function studies are based on the analysis of at least one left ventricular angiogram at a 30° right anterior oblique projection. For each ventriculogram measurements, a sinus beat and a post-extrasystolic beat are obtained for the establishment of the contractile reserve of the ventricle. An expert angiographer, blind to treatment allocation and patient progression, will draw the end-diastolic and end-systolic silhouettes using the CASS system. The left ventricular volumes and LVEF are determined and the regional wall motility is analysed. Sheehan’s method [38] is used to study asynergy, dividing the superimposed silhouettes by 100 radii of systolic wall shortening, from end-diastole to end-systole. The abnormal contracting segment is defined as the percentage of radii showing akinesia or dyskinesia. The mitral valve function is evaluated and, when present, mitral insufficiency is graded according to severity.

### Echocardiographic measurement of ventricular function

Echocardiography is used as a secondary endpoint for the purpose of comparison with published trials, as well as to provide additional non-invasive time points of LVEF evaluation. LV function will be measured with echocardiography at baseline (visit 1) as well as 6 (visit 5), 12 (visit 6), 18 (visit 7) and 24 (visit 8) months after infusion, using standardized imaging protocols to calculate ejection fraction using the Simpson’s rule. Although a discrete positive correlation has been described among LV angiography and echocardiography estimates [39], LVEF measurements by various techniques are generally not interchangeable [40]. However, LVEF changes trends measured with LV angiography will be compared with those obtained using echocardiography, and the intra-subject correlation among both LVEF estimates will be analyzed in order to stablish a correlation that may help explain the long and short-term effects of BMMNC.

### BMMNCs biological characterization

To analyze biological predictive factors of response to treatment [27], aliquots of the cellular suspension infused to experimental group patient are obtained for the following purposes:

1. Automatic cell count for evaluation of total infused cellularity.
2. Immune phenotype characterization: BMMNCs aliquots (1–5 × 10^5^ cells) are stained with fluorochrome-conjugated monoclonal antibodies (FITC, PE and APC) against human cell surface markers CD34, CD133, VEGFR2, CXCR4, CD90, CD38, CD117, HLA-DR, CD45, CD105, CD73, CD14. After incubation for 15 min in darkness, BMMNCs are washed with PBS and centrifuged for 5 min at 1800 rpm. The cell pellet is then resuspended in PBS, stained with Propidium Iodide (Miltenyi Biotec) to measure cellular viability as analyzed with MACSQuant cytometer (using MACSQuantify software for quantitative measurements).
3. Cell chemotactic ability: BMMNCs migration in response to VEGF (R&D Systems, Inc., Minneapolis) and SDF-1 (R&D Systems, Inc., Minneapolis) was assessed using 24-well culture plates and 8 μm pore size inserts (BD Biosciences, Erembodegem, Belgium). A dose of 5 × 10^4^ cells is placed in the system using serum free media RPMI-1640 (BioWhittaker® LONZA, Verviers, Belgium) and 100 ng/ml of VEGF, 100 ng/ml SDF-1 or nothing (control well). After 24 h of incubation at 37 °C, cell migration is quantified using a Neubauer chamber.

### Concomitant medication

All patients must be treated with optimized conventional cardiac insufficiency medication for at least 6 months before the inclusion date and throughout follow-up, involving the use of angiotensin-converting-enzyme inhibitors, angiotensin II receptor blockers, beta-blockers, aldosterone antagonists or other diuretic drugs, potassium supplements etc. Additionally, those patients with ejection fraction lower than 30% may also receive digoxin. Treatment should be optimized following medical judgement according to patient’s symptoms/tolerance (NYHA functional class) and/or BNP levels.

Although the combination of cell therapy with G-CSF has been proven efficacious in previous reports [30], this trial has been designed to evaluate solely the effect of BMMNCs, without any stimulating factors that may interfere with the findings.

However, no other therapy to improve the ventricular function in a mechanical way, as resynchronisation therapy or cardiac transplantation, will be used during follow-up. However, non-responders to a previously implanted defibrillator can be included. In the pilot study performed by our group an inverse correlation was found among basal high-density lipoprotein (HDL) serum levels and LVEF improvement (r = − 0,41, *p* = 0,03) [32]. Consequently, patients with a lower baseline values of HDL could attain a higher improvement in LVEF. On this account, patients included in the study should not be treated with statins affecting HDL blood levels (rosuvastatin, pitavastatin and atorvastatin) 2 months prior to inclusion, as well as throughout follow-up. A wash-out period of two months is established for patients under any prohibited medication prior the inclusion in the study.

### Sample size calculation

A sample size calculation was performed based on the clinical trial study already completed by our group [32]. In this study, a mean increase in ejection fraction of 9.4 ± 9.9% was observed. At the end of the follow-up, mean values of LVEF were 38% among responders, and 27% among non-responders. The sample size calculation was done using GRANMO (v7.12) for paired or repeated measures in two groups. Accepting an alpha risk of 0.05 and a beta risk of 0.2 in a two-sided contrast, 17 subjects are required in the first group and 34 in the second one to detect a difference equal to or greater than 9 units. It is assumed a common standard deviation of 10 and a correlation coefficient between the initial and final measurement of 0.5. A loss to follow-up rate of 10% has been estimated. However, an interim analysis has been planned in order to re-calculate trial sample size based on the mean increase in ejection fraction observed in the first 20 patients that complete a 6 months’ follow-up period.

### Randomisation and blinding process

Randomisation is centrally performed by the sponsor, stratified by age (> or ≤ 45 years old) in a 2:1 block randomisation allocation BMMNCs or placebo. As sample size is small and both planned interim analysis may require a balanced distribution of the subjects within both study groups, block randomization is applied. Randomization list was computer generated by the Sponsor, and allocation is kept concealed for the research teams recruiting patients.

Investigators and patients (as well as clinical monitors) are blind to the treatment assigned. In the case of SAE, when the patients’ management could be improved by the knowledge of the blinded treatment assigned, the principal investigator may request the sponsor formal permission for subject unblinding. Additionally, specific unblinding of patients allocated to the control group may be authorized by the sponsor to allow for the treatment through compassionate use with their own previously cryopreserved BMMNCs.

### Data management and statistical analysis

Clinical data is manually registered using a paper Case Report Form (CRF). CRF data is entered in the study database using an Electronic Data Capture (EDC) system. After double data entry, resolution of all inconsistencies, and coding by means of medical dictionaries, a final quality control process will be applied. In case of compliance, the database will be considered error free and will be frozen for the statistical analysis of the data. Sponsor and investigator will have access to the final trial dataset.

A descriptive analysis will be performed including all demographic variables collected, as well as baseline clinical data. Qualitative variables will be summarized using absolute frequencies and percentages, while quantitative variables will be described using the mean, median, standard deviation, maximum, minimum and number of observations.

The efficacy evaluation of quantitative parameters will be performed by comparing basal values (prior to the administration of the product) with those recorded in the follow-up visits, using the paired Student’s t-test (if the variable meets the normality requirements), or the Wilcoxon paired test for all other cases. For qualitative parameters, the Chi-square test will be used.

Two interim analyses will be performed during the study. The first one, to ensure the statistical power of the study, will be performed when the first 20 patients reach a minimum follow-up of 6 months, and will include a new sample size calculation based on these data. In the second one, the main variable under study will be analysed once 6-months of follow-up have been completed in all patients recruited. If the results are positive, patients randomised to the control group will be treated via compassionate-use basis with their own cryopreserved BMMNCs, as early mentioned. Per-protocol analysis (PPA) will be used for all efficacy assessments. All p-values and confidence intervals will be calculated and evaluated using a 95% two-sided confidence level.

Safety analysis is aimed at estimating the number and percentage of patients dropped out due to AEs, the number of patients who have experienced at least one AE, the most frequent AEs and the number of patients who experienced at least one SAE. The 95% confidence intervals will be also determined. Safety evaluation will be performed with intention-to-treat analysis.

### Data monitoring

A clinical trial monitor, appointed by the sponsor, periodically supervises the study progress in each participating site to ensure patient rights and well-being are safeguarded, that the protocol, ethical requirements, Good Clinical Practice (GCP) standards and applicable regulations are being followed, that the necessary documentation is available and that collected data reflect exactly data recorded on the CRF. External auditors are regularly appointed by the sponsor for independent supervision of trial activities and conduct.

An independent Data Safety Monitoring Committee (DSMC) has been created as an advisory board to guarantee correct patient enrolment and withdrawal, to counsel on safety matters in interim analyses as well as throughout the duration of the trial, to provide advice on statistical issues and to be involved in any decision ensuring the compliance of GCP, protocol procedures and applicable regulations. This committee includes 4 members, independent from sponsor or clinical teams, including 2 clinical advisers in the field of cardiology, 1 clinical methodologist, and 1 expert in ethical issues.

### Safety and adverse events assessments

In accordance with GCP guidelines, all AEs and SAEs that may occur during the clinical trial (observed by the investigator or reported by the patients) whether or not attributed to the investigational medicinal product, are closely monitored until resolution or stabilization, and registered in the study CRF. Also, investigators report to the sponsor all SAEs within the first 24 h from awareness of the event. AEs will be classified on the basis of the Medical Dictionary for Regulatory Activities (or MedDRA) terminology and summarised for each treatment group and relationship to the intervention. Causality of AEs with the study intervention is assessed by the investigator, and re-evaluated by a person responsible for pharmacovigilance appointed by the sponsor. Furthermore, trial DSMC may review accumulated safety data, when appropriate, and for advising the sponsor concerning the continuation, amendment and termination of the trial.

The clinical trial will be interrupted, if any of the following conditions arise: acute arrhythmia in 3 or more patients randomised to the experimental group, severe toxicity as per WHO scale (> grade 3) related to the infusion of BMMNCs in 3 or more patients, major infections caused by the extraction or infusion procedures in 3 or more patients or mortality related to the infusion of the cell product in 2 or more patients. Trial can also be prematurely ended if the sponsor finds a reasonable cause such as: publication in the scientific literature of unacceptable risks or AEs for patients; or a similar study showing consistent results of no significant health benefits; insufficient commitment by the coordinating or principal investigators; or substantial modifications or suspension of the development of the drug under investigation.

### Ethics and dissemination

The study protocol has been approved by the Andalusian Coordinating Ethics Committee for Biomedical Research (*Comité Coordinador de Ética en Investigación Biomédica de Andalucia, CCEIBA*) and authorized by the AEMPS. All substantial amendments to the original protocol or related documents also obtained further approval from the AEMPS and the corresponding Ethics Committee (CCEIBA and *Comité de Provincial de Sevilla*). The trial protocol has been subject to seven amendments so far, including minor changes in patient eligibility criteria, participating sites and follow-up evaluations. The present manuscript describes the current approved protocol at the time of submission: Amendment 7, 16th March 2018. Neither the researchers nor the sponsor can perform any modifications to the protocol without the authorization of both the Ethics Committee and the AEMPS.

The study is being performed in accordance with the protocols and the sponsor’s standard operating procedures (SOPs). Sponsor and investigators ensure that this trial follows the recommendations of the Declaration of Helsinki (World Medical Association 2013), the International Conference Harmonization Guidelines (ICH) for GCP (CPMP / ICH / 135/95) and the current Spanish legislation in the field of Clinical Trials.

In order to guarantee the patients’ confidentiality, data generated during the study is treated in accordance with the 3/2018 Organic Law on the Protection of Personal Data and Securing of Digital Rights, Regulation (EU) 2016/679 on the protection of personal data, as well as other regulations implementing it. All personal data is kept at the hospital, and only the investigator, the trial monitor, trial auditors/inspectors, and the Ethics Committee have access.

The drug under research is considered a cell therapy medicinal product (or placebo) as defined in Directive 2001/83/EC1 as amended by Commission Directive 2009/120/EC. The sponsor of the clinical trial is holder of an insurance policy to cover for any patient who may suffer harm from trial participation. All remaining biological samples will be destroyed after the study has ended and its results have been published. Likewise, all cryopreserved tissue samples will be discarded after all patients allocated to placebo have had the right to opt for treatment (when applicable).

The sponsor and investigators are responsible for the publication of the trial results in accordance with applicable regulations to clinical trials. All items from the WHO Trial Registration Data Set were registered in the publicly accessible databases ClinicalTrials.gov (NCT02033278, January 10, 2014) and the EU Clinical Trial Register (EudraCT number: 2013–002015-98). Dissemination of results will be mainly focussed on publications in peer-reviewed scientific journals as well as presentations at national and international scientific meetings. The SPIRIT statement [41] has been observed in the publication of this study protocol and the CONSORT [42] guidelines will be guaranteed when publishing the study results in clinical journals and conferences. Trial results will also be disseminated to the public through a lay summary of results uploaded onto EU database, as well as patient congresses whenever possible.

## Discussion

DC is a heart muscle condition affecting up to 1 in 250 individuals, the majority of which are idiopathic [43]. The disease is associated with adverse outcomes, including a 20% 5-year mortality related with heart failure. Recent studies have shown that intracoronary infusion of autologous BMMNCs has a positive impact in the cardiac function of affected patients [22–29, 32, 41], but until now there are not reported studies assessing the safety and efficacy of high dose BMMNCs (5–10 × 10^8^ cells), for a follow-up period longer than one year. Other published trials evaluating the use of BMMNCs in idiopathic DC have a limited sample size, are not controlled or blinded. The present study has been designed as a randomized double-blinded and placebo-controlled clinical trial for comparing the functional recovery of patients receiving the conventional treatment for DC plus an intracoronary infusion of BMMNCs or placebo. Therefore, individuals allocated to the placebo group, as well as those receiving BMMNCs, are receiving the best standard pharmacological treatment available and are not subject to any additional risk of serious or irreversible harm, while it is possible to evaluate the true added benefit (or risk) of the BMMNCs therapy.

Patient recruitment in this trial has been proven difficult so far, due to a very low prevalence of the disease, approximately 1:250, as estimated by Hershberger et al. [43], as well as the specific clinical profile that demands the trial’s protocol. Also, this is a publicly-funded clinical trial promoted by academic clinical researchers and public health entities, which hinders the possibility to manage a larger trial with more recruiting sites. Therefore, study has been designed as a small size trial with enough power to detect a change in LVEF equal to or greater than 9%, which is considered a clinically significant difference in terms of symptomatology. However, an interim analysis is planned to allow for an adaptive sample size re-calculation based on the data from the 6 months’ post-infusion period of the first 20 patients included.

LVEF improvement is the main study variable, and it will be evaluated angiographically by an expert angiographer blinded to treatment allocation and patient’s progression. Although this technique is not the gold standard for LVEF estimation, the approach will reduce inter-observer variability and provide more accurate, repeatable results, avoiding some of the limitations of echocardiographic estimates. Cardiovascular Magnetic Resonance (CMR) was not a feasible option in the context of this study due to the high frequency of devices (Cardiac Resynchronization Therapy Defibrillator or CRT-D) in this population that interfere with CMR scans results. Also, not all participating sites had equivalent CMR techniques, which may have provided LVEF estimates based on non-comparable images, leading to increased variability of results. Consequently, the main limitation of the present trial design is its comparability across studies that measure LVEF using magnetic resonance imaging.

In order to reduce the risks of left ventricular angiography, only two assessment points are stablished throughout follow-up (at baseline and 6 months after treatment). Also, the risk of contrast-induced acute kidney injury is minimized by the exclusion of patients with serum creatinine higher than 2,5 mg/dL. However, as the moment of highest benefit (if any) after cell infusion is still unclear, complementary non-invasive measurements of LVEF using 2D echocardiography are set out at baseline, as well as at months 3, 6, 12, 18 and 24 after treatment. Although both estimates will not be interchangeable, a correlation may be stablished that will allow for drawing conclusions about the long and short-term effects of BMMNC.

In conclusion, the results of the present trial are expected to envision a new therapeutic alternative for the treatment of idiopathic DC. Current pharmacological treatments aim to improve blood flow and prevent further damage to the heart, but they are not curative. Stem cell therapy constitutes a promising alternative to palliative care, restoring cardiac structure and function. Once the safety and efficacy of this advanced therapy product is established in humans, the final goal will be to confirm these observations in future phase II and/or phase III clinical trials involving a larger number of subjects.

## Data Availability

Data availability is not applicable to this article as no datasets were yet generated or analysed.

## Abbreviations

AEMPS: Agencia Española de Medicamentos y Productos Sanitarios
AEs: Adverse events
AIAT: Andalusian initiative for advanced therapies
AMI: Acute myocardial infarction
BM: Bone marrow
BMMNC: Bone marrow-derived MonoNuclear cells
BNP: Brain natriuretic peptide
CCEIBA: Comité Coordinador de Ética en Investigación Biomédica de Andalucia
CMR: Cardiovascular magnetic resonance
CRF: Case report form
CRT-D: Cardiac resynchronization therapy defibrillator
DCs: Dilated myoCardiopathies
DSMC: Data safety monitoring committee
EDC: Electronic data capture
GCP: Good clinical practice
G-CSF: Colony-stimulating factor
HDL: High-density lipoprotein
ICH: International conference harmonization
LVEF: Left ventricular ejection fraction
MACEs: Major cardiac events
MedDRA: Medical dictionary for regulatory activities
NT-proBNP: N-terminal fragment of pro-brain natriuretic peptide
NYHA: New York heart association
SAEs: Serious adverse events
SOPs: Standard operating procedures
